# Opportunities and challenges for implementing cost accounting systems in the Kenyan health system

**DOI:** 10.3402/gha.v9.30621

**Published:** 2016-06-28

**Authors:** Elesban Kihuba, Adrian Gheorghe, Fiammetta Bozzani, Mike English, Ulla K. Griffiths

**Affiliations:** 1 Wellcome Trust Research Program, Kenya Medical Research Institute, Nairobi, Kenya; 2 Ministry of Health, Nairobi, Kenya; 3 Department of Global Health and Development, London School of Hygiene and Tropical Medicine, London, United Kingdom; 4 Nuffield Department of Medicine, University of Oxford, Oxford, United Kingdom

**Keywords:** cost accounting, unit cost, low- and middle-income countries, Kenya, costing, health information systems

## Abstract

**Background:**

Low- and middle-income countries need to sustain efficiency and equity in health financing on their way to universal health care coverage. However, systems meant to generate quality economic information are often deficient in such settings. We assessed the feasibility of streamlining cost accounting systems within the Kenyan health sector to illustrate the pragmatic challenges and opportunities.

**Design:**

We reviewed policy documents, and conducted field observations and semi-structured interviews with key informants in the health sector. We used an adapted Human, Organization and Technology fit (HOT-fit) framework to analyze the components and standards of a cost accounting system.

**Results:**

Among the opportunities for a viable cost accounting system, we identified a supportive broad policy environment, political will, presence of a national data reporting architecture, good implementation experience with electronic medical records systems, and the availability of patient clinical and resource use data. However, several practical issues need to be considered in the design of the system, including the lack of a framework to guide the costing process, the lack of long-term investment, the lack of appropriate incentives for ground-level staff, and a risk of overburdening the current health management information system.

**Conclusion:**

To facilitate the implementation of cost accounting into the health sector, the design of any proposed system needs to remain simple and attuned to the local context.

## Introduction

The adoption of cost accounting systems in the health sector gained momentum in the late 1980s as a pivotal step in health financing reforms (1–3). Such systems collect clinical, financial, and human resources data from health service providers across multiple health information systems (HIS) and summarize the data in monetary terms based on a set of predefined units of costing (4). Evidence from high-income settings shows that cost accounting systems can be effectively used to calculate national, sub-national, and facility-level average unit costs (5). The resulting economic information, be they for final (e.g. average cost per tuberculosis patient treated) or intermediary products (e.g. average cost per inpatient day), are further used to inform decisions regarding price-setting, provider reimbursements, and cost control initiatives (4).

A recent review limited to high-income countries identified substantial transnational differences in health sector cost accounting practices in terms of data collection (scope, frequency, data sources, and validation rules) and methodology (e.g. structure of cost centers and approaches to valuation) (6). For example, data collection for costing purposes can occur annually (e.g. England, Germany, and the Netherlands), every 1–2 years (Australia) or on an irregular basis (e.g. Austria and Italy). In some countries, participation in the costing exercise is mandatory for all contracted providers (e.g. England and Medicare in the United States), while in others only a selected group of providers contribute data (e.g. Finland and France). Similarly, cost accounting regulations for providers can be prescriptive (e.g. the mandatory model in England) or flexible (e.g. in Australia a range of cost allocation methods are available to calculate and report hospital-specific costs). Irrespective of the cost accounting design, its implementation appears to rely on two main factors: the application of information technology to ensure data collection and quality; and a comprehensive regulatory framework and methodology to ensure uniformity across contributing providers (6).

Health financing reforms now beckons for low- and middle-income countries (LMICs) following a call for universal health coverage (UHC). However, limited resources build pressure for accountability and efficiency (7, 8). As such, these countries require high-quality and context-specific cost of care and clinical data in order to inform advances on two of the three dimensions of UHC: service provision and financial coverage (8). However, health systems in LMICs often have weak or non-functional cost accounting systems (9). Costs, for instance, are not automatically linked with clinical activity data (10). Medical supplies and equipment are generally delivered in a top-down manner to health facilities without sharing information (11). The absence of reliable costs and resource usage data leads to practical challenges during costing exercises (12) and to a lack of transparency over the role of economic considerations in health sector decision making (13, 14). Despite this, global and national efforts to improve health data generally focus on clinical information and rarely on systems for tracking health care resources use and their associated costs (15). The generation of costs data is often externally driven, laborious and limited to a specific intervention (16). In countries where costs are available from micro-costing studies, the variation in estimates among health facilities and the lack of regular updating tends to limit their usefulness (17, 18).

Like many LMICs, Kenya lacks a sound cost accounting system despite the call for UHC to set in motion the reorganization of health care delivery. National Health Accounts (NHA) provide information on sources of health resources and program costs estimates. The data is often limited to the major conditions and does not provide economic data for specific intervention (19). Timely and quality economic data are essential to inform management control processes such as planning, budgeting, price setting, and evaluation of performance. Reliable costs data would also help decision makers in designing policies on provider payment mechanisms and costs control. Given the benefits of improving the quality of cost data, the question arises as to whether cost accounting systems can be streamlined into existing HIS in Kenya. A streamlined system would ideally produce and regularly update a basic dataset of resource use indicators as well as cost estimates on clinical procedures.

Our aim was thus to explore the feasibility of developing a cost accounting system in Kenya, which would build on existing routine health data. The paper focuses on a cost accounting system as a budgeting and costing tool, as this is what countries that have adopted some form of cost accounting system in the health sector use it for (5, 6). We start with an overview of the Kenyan health system. Then, we examine the suitability of routine health data and the data architecture for costing purposes, followed by an exploration of organizational factors and their implication for implementation. The paper ends with a proposed option for advancing cost systems in Kenya informed by our analysis.

### An overview of the Kenyan health system

The new constitution, which came into force in 2013, provides for two levels of government: a national government and a decentralized level consisting of 47 counties. Following the reform, an act of parliament and a new health policy framework were formulated to bring the provisions of the constitution touching on health matters into force (20, 21). According to these, national government is responsible for leadership, technical support, and policy development in the health sector, while the 47 county governments are responsible for health services provision. Both levels have a shared role of planning, budgeting, and coordination. Sequential five-year strategic and investment plans are laid out to achieve policy objectives. In addition, it is mandatory for health departments and facilities to develop and implement annual operating plans and budgets.

Health services are provided by a mix of public and private health facilities, and are structured hierarchically into three levels: county primary care facilities, county referral hospitals, and national referral hospitals. Primary health facilities include dispensaries, health centers, and private clinics providing outpatient and preventive services. County referral hospitals provide a range of curative services while the national referral hospitals comprise units that provide highly specialized services. Approximately 62% of the total number of health facilities are publicly owned (22).

The health system is predominantly financed by government schemes, which account for 41% of total health expenditures. At least 17% of the population has some form of health insurance. Out of pocket and not-for-profit institution sources account for 27 and 21% of total health expenditure, respectively (19). The lion's share of these resources is spent on recurrent items such as equipment, medical supplies and human resources (23).

The achievement of UHC is an explicit goal for the Kenyan health system (21, 24). A fund established in 2013 reimburses maternal and child health services provided at public health facilities. The National Hospital Insurance Fund (NHIF) benefits package, a social health insurance scheme, has also been expanded to cover outpatient services.

## Methods

### Conceptual framework

We applied the Human, Organization and Technology fit (HOT-fit) framework to evaluate the feasibility and options for implementing a cost accounting system in Kenya (25). This framework has been validated in a number of HIS case studies (25–27). The HOT-fit model can be used to analyze the linkages between HOT factors and thus assist in understanding information system requirements in complex health system environments. Table 1 presents a list of the dimensions and related themes derived from the framework.

**Table 1:** Outline of dimensions and key themes covered

Domains	Dimensions	Themes
Technology	Data flows	Current flow of routine health data
	System quality	Integration, reliability, flexibility, usefulness
	Information quality	Usefulness, reliability, accuracy, and completeness
Organizational	Environment	Policy framework
		Managerial autonomy
	Structure	Budgeting and accounting
Human	Implementation approaches	Implementation experience with data repositories
	Technical capacity	Human resources for costing

### Data

Data were collected through a number of qualitative methods, namely a document analysis, stakeholder interviews, and observations.

An analysis of 39 government policy documents on health system and health information was carried out to understand the context, scope, and implementation experiences in Kenya. We identified the documents following a hand search of the websites of key Kenyan institutions and engaging key health experts. The review process involved multiple readings, notes taking, and coding to address the defined themes.

Findings from the review were supplemented by 32 key informant interviews conducted between February and July 2014 (Appendix 1). The number and types of included organizations represent the main actors involved in the development of HIS in Kenya. Key informants were purposively selected based on their knowledge of the sector. Interviews were conducted using an interview guide (Appendix 2) in which defined themes were covered, including how clinical data and health resource usage are tracked in the public health sector, existing constraints, required improvements as well as respondents’ thoughts and attitudes toward further developments of existing systems. Two researchers took notes independently during each interview and consolidated their findings afterwards. Notes taken during the interviews were kept confidential. The interviews were not recorded.

Field visits were conducted to four counties (Nairobi, Murang'a, Nakuru, and Kajiado), where data management, data flow, and budgeting activities at both county finance offices and health facilities were described and demonstrated. Overall these counties account for 17% of the total population and 11% of the total number of health facilities (28). Inclusion criteria for selecting the sites were availability or plans to introduce an electronic platform for health data, either at the county level or at selected facilities, as well as ease and security of access and feasibility within budget and time. The sites were studied intensively to generate adequate description of data flow and budgeting activities.

We applied the framework approach to interview data, which is suitable for data management and analysis when a study is policy oriented (29, 30). The emerging patterns following charting of interview data summaries were mapped to the predefined themes. The descriptions and explanations generated were triangulated with document analysis findings and data from field notes based on themes adapted from the HOT-fit conceptual framework (Table 1) in order to generate a description of the HIS. The overall quality of information systems in Kenya was assessed under the following themes: integration, reliability, flexibility, usefulness, and completeness. The health system's capacity to support cost accounting was analyzed by considering the existing policy framework, the degree of local autonomy, implementation experiences, and human resources capacity.

The study received ethical approval from the Kenya Medical Research Institute (KEMRI) ethics committee (Ref KEMRI/RES/7/3/1) and the London School of Hygiene & Tropical Medicine (LSHTM) ethics committee (Ref 7235).

## Results

The results are presented in accordance with the defined themes, illustrated with quotes from the documents and interviewees. Table 2 presents a summary of results by themes.

**Table 2:** Fit among the technology, organization and human components

Fit	Lack of fit
**Technology components**	**Technology components**
Data repositories for health data are in place.	Data repositories at local and higher levels are not interconnected.
Uptake of information technology in the sector is on the rise.	EMR use is restricted to large hospitals and outpatient departments.
DHIS2 and EMRs modules are highly customizable.	Poor information systems auditing practices.
Patient-level data in source documents on resource usage and services provided is readily available.	Data accuracy for reported aggregate clinical data is poor.
**Organizational components**	**Organizational components**
Broad social and economic goals are positive.	Lack of framework to guide costing process.
Political will.	Providers lack managerial autonomy.
There is pressure for introducing managerial control practices in the health sector.	No strategic budgeting and pricing.
Activity based funding and provider payment systems have been proposed for adoption.	
**Human components**	**Human components**
Rich implementation experience with data repositories.	Data systems were implemented using external funding.
	Chronic shortages of records officers and statisticians.
	Lack of appropriately trained staff for costing.

Source: Authors.

### Technology factors

#### Description of flow of routine health data

**Fig. 1:**
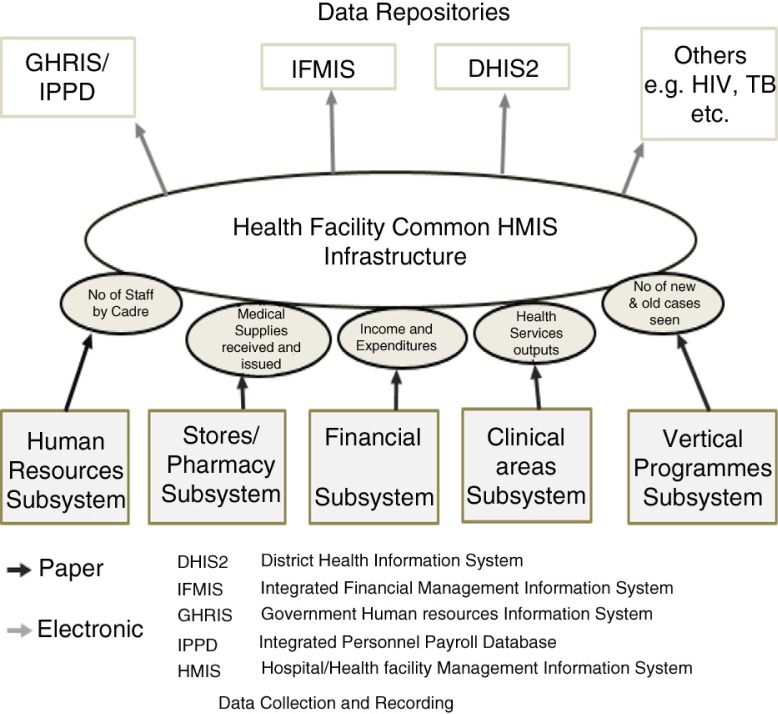
Kenyan Public Health Management Information System (HMIS). Source: Authors.

Health facilities are the nexus of the HIS architecture (Fig. 1). At this level, clinical data are generated during patient care and administrative data are generated as a by-product of managing health resources. Due to the multiplicity of data sources, the facilities’ health management information systems (HMIS) tend to be complex and comprise a mix of independent sub-systems. Each sub-system in the HMIS has a similar structure: a paper-based data collection tool or register and a data reporting tool which identifies the data elements for reporting. Overall, thousands of data elements organized into 16 registers or data collection tools, 14 reporting tools, and 4 tally sheets have been defined and adopted in the health sector. Data collected by these sub-systems are aggregated periodically and transferred to the district, county, or national level to a number of data repositories reporting clinical, human resource, and financial data. In recent years, parts of this process have been digitalized at several public hospitals across the country, as a host of electronic medical records (EMR) systems were implemented.

Clinical data are reported through the District Health Information System (DHIS2), which supports the analysis of aggregate health data including socio-demographic indicators, epidemiologic data (e.g. number of confirmed malaria cases), health services outputs (e.g. number of vaccinated individuals), and resource use (e.g. medicine stocks) (31). The DHIS2 provides for various categories of registered users, such as health workers, health managers, records officers, and even the general public. In addition to DHIS2, other systems collect information on clinical activity for different purposes. The most comprehensive of these are the Kenya HIV/AIDS Program Monitoring System (KePMS) and the NHIF information system, which tracks social insurance claims data.

Health workforce in Kenya is tracked across three systems: the Kenya Health Workers Information System (KHWIS) developed by an independent NGO (32); the MoH-operated Regulatory Human Resources Information System (GHRIS); and the Integrated Personnel Payroll Database (IPPD) (33).

Financial information is tracked using the Integrated Financial Management Information System (IFMIS), introduced from 2007 in all public institutions to track expenditure data (34). In addition, the Electronic Project Monitoring Information System for Kenya (e-ProMIS) was introduced in 2009 by the Treasury to track, monitor, and evaluate public sector projects throughout their life cycle. Other parallel financial monitoring systems serve particular funding streams, such as the Health Sector Services Fund (HSSF) (35).

#### System quality

The automation and integration of health data management systems have been high on the policy makers’ agenda. The Ministry of Health (MoH) Strategic and Investment Plan 2013–2017 emphasizes the need to interlink data repositories to ensure information systems are inter-connected and web-based if possible (22). It is now a requirement for any additional information system to support integration with the DHIS2. In fact, the government aims to develop a common data architecture by promoting the ‘use of defined standards for exchange of patient and aggregate-level data across information systems’ (36). While there are plans to achieve further integration between DHIS2 and other relevant information systems, such as KHWIS, IFMIS, and EMR systems, the interoperability of systems and data remains a major challenge. A key obstacle could be the ‘multiplicity of databases by various users within the Sector’ (23). Indeed, interviewees also noted that there have been few achievements to date as there is ‘no structure’ nor capacity responsible for integration within the MoH.

The *reliability* of a cost accounting system would be dependent both on the integration with related systems and on the state of the relevant infrastructure. The uptake of information technology in the sector is on the rise. Government documents report that all hospitals have internet connection (28) and approximately one third of the hospitals have adopted some form of EMR system (37). However, the infrastructure outlays in the health centers and dispensaries paint a different picture. These facilities are less likely to afford EMR infrastructure and only 16% (1,201/7,388) have access to internet services (28). Moreover, due to their rural location, they are often exposed to fluctuating electricity supply. The field visits confirmed that it is common practice for DHIS2 information to be recorded manually at low-level facilities and taken to county health offices, for data entry, leading to delays and increasing the risk of error. Generally, we found no evidence that ICT infrastructure had been committed for cost accounting purposes in the facilities we visited.

*Flexibility* is a key feature of current HIS developments in Kenya. DHIS2 modules are highly customizable, e.g. new indicators and tools can be added. Similarly, developers working on a county electronic health record system with support from the World Health Organization informed us that it is possible to incorporate a financial accounting module in the system, which could inform cost calculations for health facilities.

### Information quality

Data currently reported through available information systems can be *useful* in informing a cost accounting system. As noted above, there are systems already in place for reporting information about procurement and supplies management, human resources management, financial management, and service delivery (36). For example, the GHRIS and IPPD system is a reservoir for data on human resources by deployment, level of qualification, and salaries, while the IFMIS contains data about revenue, tender prices, and expenditures.

In terms of *reliability*, auditing practices across the available information systems are highly variable. For example, DHIS2 has built-in validation rules and data checks, but is not subject to an auditing schedule. However, an audit done in 2014 is indicative of discrepancies between clinical data in source documents and reported estimates (38). On the contrary, IFMIS is regularly audited and believed to demonstrate high data validity. The auditing practices of EMR systems remain unknown, as they vary in scope and are subject to county- and hospital-level practices.

Although some of the financial data are of good quality, they cannot be used to generate unit cost estimates. The billing data used in the financial systems of health facilities show the amount paid out of pocket for each specific procedure. However, in the interviews, it emerged that these user fees are not based on actual costs but rather they are determined internally by each county. Similarly, the current reimbursement rates for maternity care, primary health care, and inpatient services by the NHIF system are not based on a cost algorithm. In addition, data on health expenditures reported into IFMIS are subject to the regular public reporting formats, which contain 32-line items. According to interviewees at the MoH finance department, this format is not aligned with the health sector discourse, where budgets should be analyzed by functions and cost centers.

Claims data collected by the NHIF system were described as ‘messy’ by an interviewee. Patient demographics and hospitalization details, which form the core data for the claim, were however said to be complete. These data were collected using electronic data collection instrument. However, claims data on services provided to and resources consumed by beneficiaries are never collected through the automated system and ‘ICD 10 coding data is incomplete’.

### Organization factors

#### Policy environment

From a *public policy* perspective, improving efficiency in services provision and resource use is an explicit objective in the sector. There is focus on three areas. First, to strengthen mechanisms for donor alignment. Second, to promote financial risk pooling mechanisms and schemes for financing delivery of services. Third, to encourage adoption of provider payment mechanisms that provide incentives for better productivity and cost-containment across the sector (22).

More specifically, the adopted 2012–2030 UHC policy recommends the adoption of a capitation payment system (24). The policy also calls for generation and use of costs data to improve payment mechanisms (24). Moreover, the sector has developed elaborate blueprints for implementation of management control systems such as performance management, zero-based budgeting, and public financial reporting (31, 34, 39, 40). However, no framework has been put forward to strengthen the cost accounting systems in the health sector to meet these needs.

In the wake of the new constitution, health facilities lack both financial and operational autonomy. Current laws require that ‘each County Treasury shall establish a Treasury Single Account’ through which payments of money to and by the various county government entities including hospitals are to be made (41). However, two out of four hospitals visited had negotiated an arrangement with the county governments that allowed them to operate their own bank accounts and manage their resources.

#### Structure of the financial system

From a financial management perspective, interviewees – particularly the representatives of international bodies – had concerns about the weaknesses of the financial system. Financial management departments have been established on paper across all health system levels, but interviewees in health facilities reported that there are few incentives to review expenditure data at this level since little financial decision-making takes place in practice.

On matters of budget development, although the recommended norm was zero-based budgeting, where resources should be allocated based on planned activities, interviewees at the county department of health reported that they are required to submit fund requests on a ‘need’ basis and when these requests ‘exceed available funds, they are revised downwards’. As such, the budgeting process is just a formality as it is neither linked to the identified plans nor is used for management of services.

With regard to procurement, interviewees reported that purchase of essential supplies and equipment is ‘carried out centrally’ by the office of the county executive officer of health. Some of the budget items paid for centrally include utilities, drugs and supplies, equipment, staff, and maintenance. Based on our observations, there is no evidence that financial data for centrally procured supplies and equipment are shared with health facilities management.

### Human factors

#### Implementation approaches

The Kenyan health system has a rich *implementation experience* with data repositories. The implementation of generic systems, which include aspects that are used for monitoring resource use (e.g. DHIS2, IFMIS, GHRIS), share a number of characteristics. First, apart from GHRIS, these systems are ‘open source’ and ‘externally funded’. Second, implementation was dominated by top-down strategies, such as mandatory reporting and policy directives. Finally, the similar structural challenges affected the implementation of all repositories. Some of the structural challenges reflected in an ICT white paper include low automation levels of operational processes, the existence of departmental silos, and disparate non-standardized data formats (42). There has been top management support to overcome these challenges at the time of implementation by training of users, simplifying the software design, procurement of ICT equipment, appointment of district champions, and piloting. For example, during the implementation of IFMIS, actions to support implementation fell under two categories, ‘ICT to support’ – aimed at providing the infrastructure and support required for a fully functional financial management system and ‘communicate to change’ – aimed at supporting change management, capacity enhancement, information generation and dissemination, education, and effective communication among stakeholders (34).

With regard to implementation experiences of EMRs, there is a concern that the level of support may be insufficient in relation to actual needs at the health facility level. This concern is worsened by persistent fragmentation of the HIS network. In addition, small hospitals cannot afford EMR implementation. For example, public hospitals are adopting proprietary, off-the-shelf EMR systems through independent efforts. Furthermore, a report evaluating EMR implementation experience in 25 hospitals in Nairobi shows that these were faced by a number of challenges, partly because there was ‘lack of consensus between senior managers and user departments’ and ‘poor planning’ (43).

#### Human resources for costing

The sector has inadequate personnel and inadequate skills development for staff. Interviewees reported that chronic shortage of ‘records officers’ was hampering data management operations. The MoH lacks adequate numbers of statisticians, health economists, health records officers, and epidemiologists who would be vital for handling data management activities (22). For example, the MoH has employed only 844 health records officers and 6 economists against a staff establishment of 4,071 and 53, respectively. As a result data management activities were largely carried out by nurses. These concerns were also echoed by informants from non-state actors who felt that the system is very ‘vulnerable’ due to shortage of human resources.

### Fit analysis

The fit among the technology, organization, and human components is summarized in Table 2. Our analysis shows that important preconditions related to economic and social goals have been met. Specifically, the push to introduce managerial practices in the health sector, such as budgeting and performance management, as well as the planned introduction of a system for reimbursement of cost of services by the MoH (24). These pressures have led to efforts to improve data availability through information technology, which can invigorate the development of a cost accounting system.

We identified a number of ‘misfits’, which require broader organizational changes. First, data repositories are not interconnected and data auditing has not been institutionalized. Second, the health sector lacks a policy framework to guide costing. Third, providers lack managerial autonomy. Finally, the sector lacks appropriately trained staff for costing and is heavily reliant on external funding for development.

## Discussion

The vision of the government is for every Kenyan to have access to quality health services. To achieve this aim, a robust cost accounting infrastructure is required to inform the government's investment choices. Our study identified a number of opportunities and strengths in relation to the viability of a cost accounting system in Kenya – in particular, durable political commitment to digitalization in healthcare, a broad and expanding HMIS infrastructure, as well as a recognized interest from policy makers and practitioners at all levels for relevant unit cost data. In addition, a number of control initiatives established by the MoH, such as performance management, activity-based funding for maternity services, and risk adjusted payment mechanisms for providers, have been shown in the literature to stimulate the establishment and growth of cost accounting infrastructure (5, 9, 44).

Nevertheless, fundamental misalignments remain regarding the motivations of various stakeholders for cost data and the regulatory framework, which have implications for design of a cost accounting system. Despite the broad recognition of the lack of cost data in the health sector, repeated calls for improvements in generation and use of costing data have had little impact. This resonates with a previous study finding that budgeting had not been institutionalized and the sector lacks quality data for monitoring budget implementation (45). This lack of cost consciousness, a failure by design, is likely to continue across all levels in the health system unless the administration introduces uniform regulation on costing so as to create a real demand for cost data (2, 6). In addition, investment in cost accounting systems will remain unattractive in the short term in the absence of strategic budgeting and pricing, as witnessed by the continued reliance on block budgets and failure to decentralize responsibility for accountability of resources to health facilities (6, 46).

Unit cost estimates require two broad pieces of information: clinical activity data (type and volume of clinical services) and cost of procedures and consumables (4, 6). Our findings suggest that data on services delivered and resource use are readily available at health facilities, albeit largely in paper-based individual patient records. The successful exploitation of information technology for data management activities represents a positive step toward improvement of the quality and availability of service data for costing. However, the implementation pattern of new technology in the country is largely determined by local availability of funds, leading to local initiatives which are disjointed and rarely subjected to quality audits. This has negative implications for the implementation of cost accounting systems that require broad interoperability and reliability (47). As such, advanced HIS with a capacity to support costing are likely to be discrete and located in large hospitals only (48).

The shortage of skilled staff and low analytical capacity for both epidemiological and financial data across all levels of the health system is of concern. Moreover, health workers in administrative posts are unlikely to be conversant with accounting language. Training, recruitment of skilled staff and task shifting could provide a more conducive environment for implementing a cost accounting system.

Past implementation experience with data generation and reporting systems shows that governance plays an important role. The government took the lead in modernizing HMIS in 2009 (49) and in all data repositories that have successfully been implemented since. In addition the MoH is involved in enforcing norms and standards for HMIS. We argue that this represents an improvement in the administrative capacity of the MoH.

Our assessment of the Kenyan context is compatible with other assessments of HMIS development in LMICs (9, 50, 51), highlighting both improvements in data quality and structural limitations related to infrastructure, capacity, and stakeholder engagement. Given the need to build cost accounting systems on the back of existing systems, the implementation of a nationwide health reporting system such as DHIS2 is instrumental to any cost accounting development. The overall performance and relevance for decision making of DHIS2 will thus ultimately determine the design and scale-up opportunities of any cost accounting initiative. Recent experience suggests that while significant HMIS advances are undoubtedly achievable when the right partnerships are forged (52), drawing a universal roadmap to cost accounting implementation remains difficult given the need to account for the country-specific institutional context.

While the study examined an underexplored subject in health system research and used qualitative methods to understand and capture essential aspects that would influence cost accounting implementation, there are a number of limitations. First, we did not interview actors located outside the country and in the private sector, who might have an interest in this area and likely to influence future development. Second, our study did not examine questions related to measurement approaches for costing or strategies that should be employed to promote uptake of costing in Kenya. Finally, the paper did not explore ways of dealing with cases where actuals costs data maybe unavailable. These unanswered questions require further research to fine-tune cost accounting implementation.

### How can a cost accounting system be best implemented in Kenya

In our analysis of implementation options, we focused on how a cost accounting system may fit in the larger HMIS landscape with respect to data collection for costing purposes. The success of implementing a cost accounting system would depend on specific incentives and context. In relation to the technical aspects, our analysis suggests that the steady development of HIS in the sector coupled with availability of data on patient transactions represents a good foundation. Moreover, the flexibility of some existing EMR platforms and DHIS2 suggest that some system requirements can incrementally be met.

We believe that a nationwide implementation would be premature at the moment because only few health facilities have the capacity to accommodate it. A more suitable way would be to implement a system in targeted facilities – the current leaders in information development – by capitalizing on their interest and ongoing developments. Two main reasons have informed our position. First, it is advantageous to build on already existing efforts. Second, the use of reference hospitals, sometimes with recruitment of less than 10 hospitals, has been demonstrated to be adequate in meeting cost data needs in some high-income countries (6). Such an arrangement could be streamlined into a comprehensive national costing exercise through regulation, which would make good use of the already available data to generate context-specific economic information. This is essential for Kenya in the exploration stage of performance monitoring and provider reimbursement reforms. NHIF and county administrations are particularly incentivized in this direction, as NHIF is considering the introduction of activity-based reimbursement informed by case mix coding. In addition, the counties would welcome a switch to budgeting for health programs instead of relying heavily on historical allocations.

## Paper context

Generating economic data to guide health reforms is a legitimate objective of the health sector. However, little effort is devoted to developing cost accounting systems for generating economic data. This paper highlights informational, organizational and human factors likely to affect implementation of cost accounting systems in Kenya. Some of the factors are supportive while others are not. Thus, the design of any proposed system needs to remain simple and attuned to the local context.

## Appendix

**Appendix 1:** List of interviewees and their host organizations

Type of organization	Organization/department	Position	No. of interviewees
National Ministry of Health	Monitoring and Evaluation	Head, M&E	1
	Health information system	Head, HIS	1
	Health Financing	Head, Health financing division	2
National Treasury	IFMIS		2
	e-PROMIS	Director	1
Social Insurance Agency	National Hospital Insurance Fund		1
University of Nairobi	School of Economics	Senior Lecturer	1
	School of Public Health	Head, Health system and policy department	1
Tertiary Hospital	Kenyatta hospital		1
Multilateral organizations	WHO	Health System Advisor	1
	World Bank	Regional Health Specialist	1
	DFID	Secretary, Health & Education departments	1
USAID or PEPFAR funded	Abt Associates		1
implementers	i-TECH		1
	Futures group		3
Nairobi County	Mbagathi District Hospital		4
Kajiado County	County administration		2
	Hospital	Health administration officer	1
Muranga County	County administration		2
	Muranga County Hospital	Medical Superintendent	1
Nakuru County	County administration		1
	Nakuru Regional Hospital		2
**TOTAL**			**32**

### *Appendix 2*. Interview Guide

The following questions should be addressed during the interviews. When needed, use the suggested probes. Please make sure to note whether answers address past or current events. Interviewees are not expected to answer all of the questions and you should use your judgment in deciding which questions are appropriate, acceptable, and reasonable for a specific interviewee.

Please start each interview with the following introductory questions:

1. What is your position?
2. How long have you had your current job?
3. What led you to seek/accept this position?
4. What are the responsibilities in your current job?
5. What responsibilities take up most of your time?
6. In what way do you work on HMIS?

**Specific questions to Ministry of Health and other government officials**

1. Please explain the annual budgeting process between the Ministry of Finance and the government health sector
  Probes:
  - When does the process start and when does it finish?
  - What type of information is the budgeting request from the Ministry of Health based on?
  - To what extent is it based on last year's request?
  - To want extent is it based on costed plans?
2. Please explain how funds are allocated to regional health authorities
  Probes:
  What type of information is the allocation based on?
3. Please explain how funds are allocated to national health programs, such as vector control, HIV/AIDS, etc.
  Probes:
  - Does each program make a request?
  - Is it largely based on the allocation for previous years?
4. Please explain to what extent funds from international partners are channeled through the government budget of health.
5. Please give an overview of your HMIS
  Probes:
  - What type of modules/parts is it divided into?
  - How does information flow from health facilities and up through the system?
  - In your view, what is the most useful part of your HMIS?
6. Please give an overview of the financial part of the HMIS
  Probes:
  - What type of information is recorded?
  - How does information flow from health facilities and up through the system?
7. Do you know of any former initiatives/projects that were established to improve your financial information system? For each, please list when they took place, the main aims, and sources of funding
8. If so, were they successful?
9. If you don't think they were successful, what do you consider the most important factors explaining the failure?
10. In what ways would you like your financial HMIS to be improved?
11. What do you think the most important constraints are for improvement?

**Specific questions to international partners**

1. Is your organization currently involved in improving the financial HMIS in the Kenyan health sector?
2. Has your organization previously been involved in projects for improving the financial HMIS in the Kenyan health sector?
3. Please summarize your current engagement in the Kenyan health sector
4. In your projects, have you established cost monitoring systems?
5. As part of your previous or current projects, have you undertaken studies to determine unit costs of health interventions?
6. Do you know of any former initiatives/projects that were established to improve financial information systems in the health sector? For each, please list when they took place, the main aims, and sources of funding.
7. If so, were they successful?
8. If you don't think they were successful, what do you consider are the most important factors explaining the failure?
9. In what ways would you like your financial HMIS to be improved?
10. What do you think the most important constraints are for improvement?

**Specific questions to management staff at health facilities**

1. Please describe how expenses of your facility are managed
  Probes:
  - What are the financial flows?
  - How are expenses paid? Directly from your facility or via regional or national offices?
  - Please list the financial flows of all main expenses
2. How do you determine your annual budget?
3. Please summarize your HMIS. This includes patient records, ICD codes, etc.
4. Are there any costs or expenditure aspects of your HMIS?
5. Have you got any system in place that tells you how much it costs to have a patient stay in the ward for one night?
6. Have you got any system in place that can tell you the costs of treating a patient for a particular illness?
7. What improvements do you think are most needed in your HMIS?
8. What improvements do your think are most needed in your budgeting and accountancy system?
9. What aspects would you like to see in an ideal financial HMIS?
